# Linking warming effects on phenology, demography, and range expansion in a migratory bird population

**DOI:** 10.1002/ece3.4746

**Published:** 2019-02-14

**Authors:** José A. Alves, Tómas G. Gunnarsson, William J. Sutherland, Peter M. Potts, Jennifer A. Gill

**Affiliations:** ^1^ School of Biological Sciences University of East Anglia Norwich UK; ^2^ South Iceland Research Centre University of Iceland Laugarvatn Iceland; ^3^ Department of Zoology University of Cambridge Cambridge UK; ^4^ Farlington Ringing Group Southampton UK; ^5^ Present address: Department of Biology, CESAM ‐ Centre for Environmental and Marine Studies University of Aveiro Aveiro Portugal.

**Keywords:** climate change, population dynamics, species distribution, temperature, waders

## Abstract

Phenological changes in response to climate change have been recorded in many taxa, but the population‐level consequences of these changes are largely unknown. If phenological change influences demography, it may underpin the changes in range size and distribution that have been associated with climate change in many species. Over the last century, Icelandic black‐tailed godwits (*Limosa limosa islandica*) have increased 10‐fold in numbers, and their breeding range has expanded throughout lowland Iceland, but the environmental and demographic drivers of this expansion remain unknown. Here, we explore the potential for climate‐driven shifts in phenology to influence demography and range expansion. In warmer springs, Icelandic black‐tailed godwits lay their clutches earlier, resulting in advances in hatching dates in those years. Early hatching is beneficial as population‐wide tracking of marked individuals shows that chick recruitment to the adult population is greater for early hatched individuals. Throughout the last century, this population has expanded into progressively colder breeding areas in which hatch dates are later, but temperatures have increased throughout Iceland since the 1960s. Using these established relationships between temperature, hatching dates and recruitment, we show that these warming trends have the potential to have fueled substantial increases in recruitment throughout Iceland, and thus to have contributed to local population growth and expansion across the breeding range. The demographic consequences of temperature‐mediated phenological changes, such as the advances in lay dates and increased recruitment associated with early hatching reported here, may therefore be key processes in driving population size and range changes in response to climate change.

## 1 INTRODUCTION

Understanding biological responses to global climatic and environmental change is among the most urgent of challenges facing ecologists and conservationists (Lebreton, 2011; Parmesan, 2006). Changing climatic conditions have been linked to changes in phenology (Parmesan & Yohe, 2003; Visser, Holleman, & Gienapp, 2006), distribution (Chen, Hill, Ohlemüller, Roy, & Thomas, 2011; Hickling, Roy, Hill, Fox, & Thomas, 2006; Hill et al., 2002), and demography (Both, Bouwhuis, Lessells, & Visser, 2006; Hansen, Aanes, Herfindal, Kohler, & Sæther, 2011; Post & Forchhammer, 2008) across many taxa (Root et al., 2003; Thackeray et al., 2010; Walther et al., 2002), but the mechanistic processes driving these changes in free‐ranging animals, and their population‐level consequences, remain poorly understood (but see Lane, Kruuk, Charmantier, Murie, & Dobson, 2012; Ozgul et al., 2010). Predicting species responses to future climatic scenarios requires a mechanistic understanding of the ecological, behavioral, and historical factors that influence species demographic and distributional responses to changing environments (Mustin, Sutherland, & Gill, 2007; Norris, 2004). Quantifying the mechanisms through which changing climatic conditions can influence changes in population size and distribution is therefore key to predicting future responses to environmental change and identifying associated conservation actions (Beale, Lennon, & Gimona, 2008; Guisan et al., 2013).

Phenological changes in response to climate change (e.g., Amano, Smithers, Sparks, & Sutherland, 2010; Gordo, 2007) will only directly affect population demography (Pettorelli, Pelletier, Hardenberg, Festa‐Bianchet, & Côté, 2007) and distribution (Van der Jeugd et al., 2009) if fitness parameters are influenced by changes in the timing of events across the annual cycle (Chuine, 2010; Visser et al., 2006). Phenological mismatches with resource availability have been linked to local population trends in several species (Both et al., 2006; Gaillard et al., 2013; Hansen et al., 2011), but the extent to which these patterns can drive large‐scale population and range expansion or contraction in free‐ranging animals remains poorly understood. In addition, recent studies have found that climate‐mediated changes in phenology may not always lead to changes in population size (Dunn & Møller, 2014), even when fitness effects are apparent (Reed, Grøtan, Jenouvrier, Sæther, & Visser, 2013a; Reed, Jenouvrier, & Visser, 2013b). Understanding how local climate effects can potentially scale up to population‐level changes requires quantification of the magnitude of climate effects on individual traits and demographic rates across a population (McLean, Lawson, Leech, & Pol, 2016).

**Figure 1:**
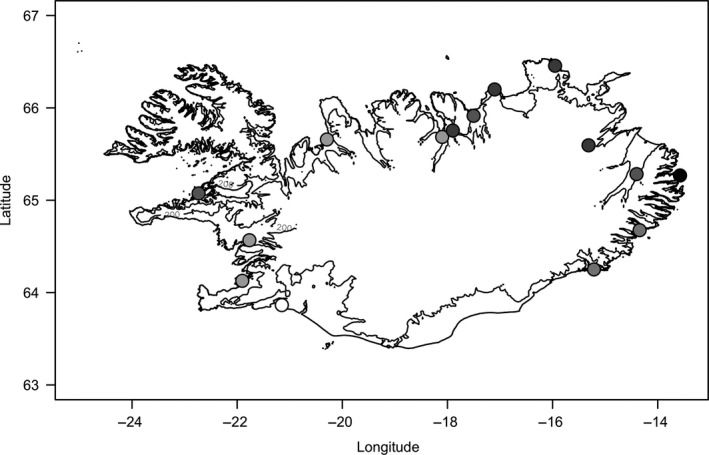
Map of Iceland with 200 m asl contour line below which most suitable habitat for breeding Icelandic black‐tailed godwits occurs. Circles show areas occupied by the species before the 1900s (white) or colonized during the 1950s (very light gray), 1960s (light gray), 1970s (gray), 1980s (dark gray), or 1990s (black). All these areas have an operational weather station collecting data for each lowland basin (see Supporting Information Table S3 for details)

Among the most rapid and severe changes in climatic conditions to have occurred over the last century are the warming temperatures in arctic and subarctic zones (IPCC, 2007; Robinson, 2009). Iceland lies mostly within the subarctic climate zone and has experienced a general increasing trend in temperatures since records began in 1845 (Einarsson, 1984; Jónsson, 2006), with rapid temperature increases in the 20th century during which annual mean temperature at the longest running weather station rose ~1.2°C (Jónsson, 2006). Iceland hosts internationally important breeding populations of many migratory bird species (Gunnarsson, Gill, Appleton, et al., 2006a), for which changing climatic conditions could have important implications. For example, the breeding and wintering range of Icelandic black‐tailed godwits (*Limosa limosa islandica*) has expanded over the last century, concomitant with this warming period (Gill et al., 2001; Gunnarsson, Gill, Newton, Potts, & Sutherland, 2005a). In the early 1900s, this species was restricted to the southern lowlands of Iceland but since then it has gradually colonized coastal lowland areas throughout the country (Figure 1) with larger areas closer to occupied sites being colonized first (Gunnarsson, Gill, Petersen, Appleton, & Sutherland, 2005b). The population now numbers ~50,000 individuals, which is likely to be an approximately 10‐fold increase in numbers over the last century (Gill et al., 2007; Gunnarsson, Gill, Potts, et al., 2005c), but the environmental and demographic changes underpinning this population increase and range expansion are not known (Gill et al., 2007). Icelandic godwits are long‐lived migratory shorebirds (Alves et al., 2012; Gunnarsson, Gill, Atkinson, et al., 2006b) with a typical lifespan of ~15–20 years (Gill et al., 2007), that breed almost exclusively in Iceland and winter in coastal zones of north and west Europe (Gill, Hatton, & Potts, 2002). They have a modal clutch size of four eggs, nesting in lowland wetlands dominated by grasses or by dwarf birch (*Betula nana*) and sedges (*Carex* spp.) (Gunnarsson, Gill, Newton, et al., 2005a) and are among the largest nest‐concealing species of the Scolopacidae (Cramp & Simmons, 1983), thus requiring a suitable vegetation height in order to initiate nesting. As the onset and rate of vegetation growth in subarctic ecosystems (Thorvaldsson, Björnsson, & Hermansson, 2005), as well as the timing of emergence of invertebrate prey for wader chicks (Halldórsson et al., 2013; Tulp & Schekkerman, 2008), are strongly temperature‐dependent, timing of nesting, and chick growth rate are likely to be influenced by local temperatures.

Over eight years during which temperatures in Iceland varied substantially, we quantified (a) the influence of temperature on nest cup vegetation heights, laying dates and the duration of the pre‐fledging period of Icelandic godwits, and (b) the subsequent influence of hatching dates on recruitment of chicks to the wintering or subsequent breeding population. We then use these relationships to investigate the potential contribution of warming‐derived changes in phenology and recruitment to the rapid population increase, by quantifying (c) variation in spring temperature and hatch dates across breeding areas colonized at different times during the 20th century, and (d) the magnitude of temperature changes and consequent estimated rates of change in godwit recruitment within these areas during the period of range expansion.

## 2 MATERIALS AND METHODS

### 2.1 Local‐scale phenology of breeding season events

#### 2.1.1 Timing of nesting and vegetation growth

The breeding success of godwits was intensively monitored annually between 2001–2003 and 2005, and 2010–2013, with 11 study sites in South Iceland being surveyed 1–2 times per week during the breeding season (Supporting Information Table S1). For each godwit nest found, the incubation stage of the clutch (all eggs) was measured using standard egg floatation method, allowing the laying date (day when the last egg was laid) and hatch date to be estimated. Categorical classification following (Liebezeit et al., 2007) was adapted to our study species (Supporting Information Table S2): firstly the average length of incubation was determined in successfully hatched nests which were found during egg‐laying (22.7 days ±0.3 *SE*,* n* = 7); then two additional floatation levels were added to the five described in Figure 2 of Liebezeit et al. (2007) to allow greater accuracy during the later stages of incubation, based on field observations (J. A. Alves, personal observation). When eggs of the same clutch varied in incubation stage, the middle value between categories (in days) was assigned to the clutch. Every nest was visited regularly, and successful nests were revisited at the estimated hatching date in order to capture and mark chicks and adults with individual combinations of color‐rings. Vegetation height around the rim of the nest cup was quantified when nests were first located, using a tape measure at four equidistant points from the center of the nest, and also at 15 randomly located points in the vicinity of the nests (within 100 m) throughout the breeding season (May to July) in 2011–2013.

**Figure 2:**
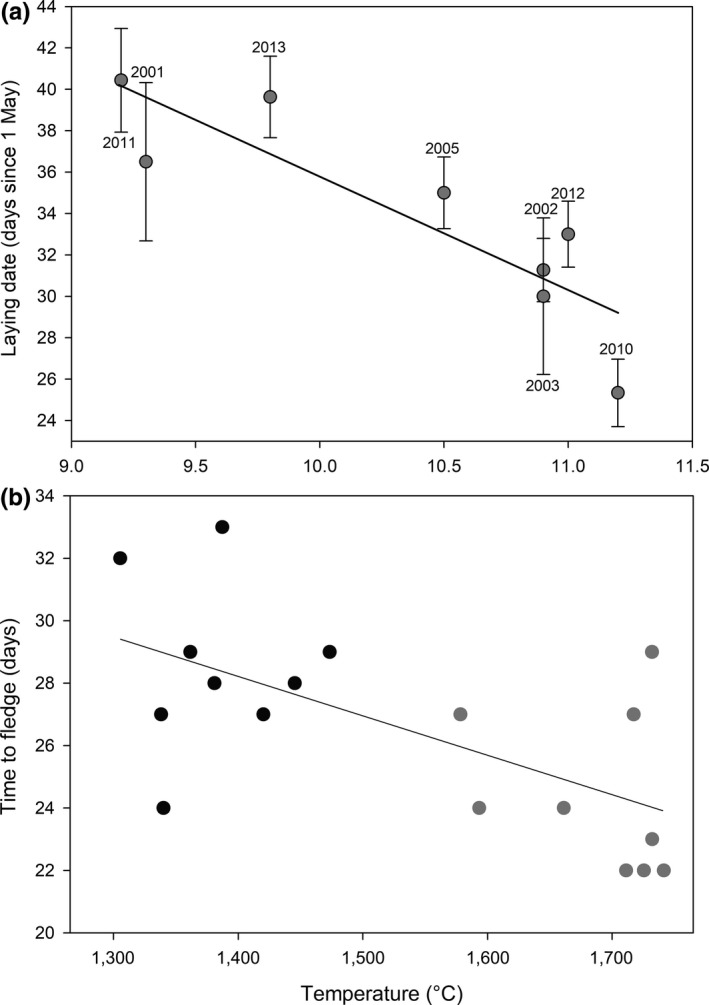
Changes in (a) mean (± *SE*) annual nest laying date (days from 1st of May); and (b) length of the period from hatching to fledging, of Icelandic black‐tailed godwits in relation to: (a) mean daily temperature in June between 2001 and 2013 (laying date = −5.49 * temperature + 90.7, *r* ^2^ = 0.75, *F*_(1,6)_ = 18.32, *p* = 0.005), and (b) cumulative daily temperature in 2012 (gray) and 2013 (black) during the 21 days post‐hatching (time to fledge = −0.013 * cumulative temperature + 45.91, *r* ^2^ = 0.40, *F*_(1,16)_ = 10.69, *p* = 0.005)

#### 2.1.2 Length of the pre‐fledging period

In 2012 and 2013, 32 godwit families were tracked during chick‐rearing, from hatching to fledging (*n* = 18) or brood loss (*n* = 14). The length of the pre‐fledging period could therefore be measured for 18 families (nine in each year). For each family, one of the adults was captured using either a nest‐trap or a hand‐held net‐gun (details in Edwards & Gilchrist, 2011) and fitted with an individual combination of color‐rings and a back‐mounted radio‐tag weighing 2.4 g with a 1.5‐month lifespan (PIP3, Ag393 Biotrack Ltd). The position of each radio‐tagged individual was recorded every two days using a hand‐held Yagi antenna and receiver (Sika, Biotrack Ltd) and a GPS (eTrex Garmin). Exact hatch dates were unknown for the two broods of adults trapped with the net‐gun, but the highly aggressive behavior of the adults (adult godwits stay very close to their chicks and defend them during the pre‐fledging period, Gunnarsson, Gill, Petersen, et al., 2005b) indicated that these had both been captured within 1–2 days of chick hatching (J. A. Alves, personal observation, 25 June & 3 July 2012). Models of the length of the pre‐fledging period were constructed both including and excluding these two families. Brood mortality was defined as two consecutive failures to relocate tagged families (at 2 and 4 days) after the last confirmed observation (when adults still displayed aggressive defensive behavior). When families were not located at the last known position, scans for tag transmissions were taken at vantage points (minimum of 3) in a triangular shape surrounding the last known position and covering a range of more than 1 km from that position (a distance that exceeds the distance over which godwit broods have been recorded moving; Kentie, Hooijmeijer, Trimbos, Groen, & Piersma, 2013; J. A. Alves, personal observation). Godwit chicks typically fledge at ~25 days (Kentie et al., 2013); however, some families fledged young prior to that age and, for these, visual confirmation of at least one of the fledged young was attained by reading its individual color‐ring combination.

### 2.2 Range‐wide demography and distribution

#### 2.2.1 Determining recruitment rates

In addition to the chicks marked at the 11 study sites (see above), Icelandic godwit chicks have been caught, measured, and individually color‐ringed at locations all around Iceland since 1999 (*n* = 966; yearly average = 69 ± 56.2 *SD*). The majority of these chicks were captured, marked, and measured (mass to nearest 0.5 g using a spring balance or a digital scale) after leaving the nest and so their age was unknown. As very few chicks are repeatedly recaptured during the pre‐fledging period, we used a previously published growth curve established for this species (Beintema & Visser, 1989) to estimate the age of each chick at capture (and thus hatch date, used in the model below) from their body mass at capture. Although uncertainty in age estimation increases with body mass (Kentie et al., 2013), the vast majority (>75%) of the chicks in this study weighed 150 g or less at ringing. A network of hundreds of volunteer observers regularly report observations of color‐ringed Icelandic godwits from across the migratory range (Alves et al., 2013; Gunnarsson, Gill, Atkinson, et al., 2006b), allowing recruitment to the wintering or subsequent breeding population (i.e., any record after the first autumn period) of the color‐marked chicks to be established. As the vast majority of godwit recruits were first recorded within their first 2 years of life (mean no. of years to first observation = 1.5 ± 2.1 *SD*,* N* = 394), comparison of chicks that recruited (observed after the first autumn migration period) or did not (never recorded since ringing) was restricted to chicks ringed up to and including the summer of 2011 and using sightings up to and including those in summer 2013. More recently ringed individuals (since 2012) that may not yet have been observed in the breeding or nonbreeding sites at the time when this analysis was executed were excluded.

#### 2.2.2 Range expansion

To explore the potential influence of temperature changes in recent decades on recruitment rates in the Icelandic godwit population, average monthly temperatures were extracted from the Icelandic Meteorological Office (available at www.vedur.is) for 14 lowland areas around Iceland in which the average year of colonization by godwits during the past century had previously been compiled (Supporting Information Table S3; Figure 1). The average year of colonization has been reconstructed by collating reports and records of new breeding species within each lowland basin provided by local residents which are encouraged by and submitted to the Icelandic Institute of Natural History (see details in Gunnarsson, Gill, Petersen, et al., 2005b; Supporting Information Table S3). None of the weather stations provided temperature data prior to 1949 (Supporting Information Table S3).

### 2.3 Data analyses

The influence of temperature on annual variation in mean godwit laying dates across the 11 study areas in South Iceland was explored in regression analyses using (a) mean June temperature (the month in which the majority of godwit clutches are laid; although some clutches were laid during the last week of May, temperatures during late May and June were very strongly correlated (*r*_s_ = 0.87, *n* = 8, *p* < 0.005) in these years) and (b) mean daily temperature during the laying period (from the laying date of the first nest to the laying date of the last nest located) in each year. The former analysis facilitated use of historical mean June temperatures in subsequent analyses while the latter ensured that the patterns were consistent with more highly resolved temperature data which are not available for the historical time series. Mean temperature was chosen as predictor of laying dates as this parameter is strongly related to the growth rates of grasses in Iceland (Thorvaldsson et al., 2005). Temperature data were recorded at the closest weather monitoring station (Eyrarbakki, 63°52′N, 21°09′W; which is within 7.5 km of all the nests found and broods tracked).

To explore the effect of temperature on the length of the chick‐rearing period, we calculated the daily cumulative temperature sum since hatching for each brood and used this as a predictor in a regression model with the length of the pre‐fledging period (days) as the response variable. Cumulative temperature was considered a proxy of time available for chicks to forage, as low temperatures often require parental brooding thus reducing foraging time. As cumulative temperatures will inevitably be higher for chicks that take longer to fledge, we restricted the calculation of cumulative temperatures to the first 21 days after hatching (the age at which the earliest brood fledged).

To estimate the seasonal effect of laying date on nest survival, we used a formulation of Mayfield's method (Mayfield, 1961, 1975) as a logistic model and a logit link function (Crawley, 2007) in which success or failure over a given number of days (as a binary variable) was modeled with the number of days over which the nest was monitored as the binomial denominator (Aebischer, 1999; Morrison, Robinson, Leech, Dadam, & Toms, 2014), laying date as a predictor and year as a random effect. For predated nests, the number of exposure days was considered to be the mid‐point between the maximum and minimum possible number of exposure days, given the timing of nest visits.

In order to investigate the effect of hatch date on the probability of recruiting into the wintering population, and as the data were too over‐dispersed for a capture‐mark‐recapture analysis, we constructed a GLMM with binomial error distribution and a logit link function (Crawley, 2007), with recruited (observed after the first autumn migration) or not (not recorded since ringing) as the response variable, hatch date as a fixed factor and year of capture as random factor. Variation in resighting probability of marked juveniles is not included in these analyses but systematic variation in resighting probability in relation to hatch date is unlikely.

Over the last century, godwits have expanded into progressively colder regions of Iceland. Regional variation in chick hatch dates were explored with an ANOVA test, and rates of spring temperature change during the population expansion in colonized areas were explored in a GLM, with mean decadal June temperature modeled as function of decade (1950s to 1990s), mean year of colonization (from Gunnarsson, Gill, Newton, et al., 2005a), and their interaction. Areas colonized by Icelandic godwits during the past century but for which temperature data were not available were not included in this analysis.

We then used the relationships established between (a) local temperature and hatch dates, and (b) hatch dates and recruitment probability, to estimate the magnitude of change in recruitment probabilities for the breeding areas during the decades following colonization, given average June temperatures in each area in each decade (see Supporting Information for details). These estimates conservatively assume that the relationships between temperature and timing of breeding season events are consistent at the lower temperatures that occur elsewhere in lowland Iceland. All analyses were performed in R 2.15.0 (R Development Core Team, 2011).

## 3 RESULTS

### 3.1 Effects of temperature on the phenology of breeding season events

Icelandic godwits lay their clutches earlier and rear their chicks faster when temperatures are warmer (Figure 2). In years with warmer mean June temperatures (~11°C, Figure 2a), laying dates are ~11 days earlier than in colder years (~9°C, Figure 2a). These patterns are also apparent when using mean daily temperature during the sampled laying periods in each year (measured as the mean daily temperature between the start and end of laying; laying date = −3.07 * temperature + 66.90, *r* ^2^ = 0.67, *F*_(1,6)_ = 12.28, *p* = 0.013). The estimation of laying dates using categorical data for large waders has a reported absolute mean (± *SE*) deviation ranging from 0.2 (± 0.2) to 2.5 (± 0.5) days (Liebezeit et al., 2007); however, there is no reason to expect systematic annual bias in this error and the measured differences in laying dates are considerably larger than the associated error (Figure 2a). Broods that experienced warmer cumulative daily mean temperatures during the pre‐fledging period also had significantly shorter pre‐fledging periods (Figure 2b). This relationship persisted when the two broods with unknown hatching date were excluded (time to fledge = −0.011 * cumulative temperature + 43.53, *r* ^2^ = 0.31, *F*_(1,14)_ = 6.28, *p* = 0.025).

### 3.2 Effect of breeding season temperatures and phenology on demography

**Figure 3:**
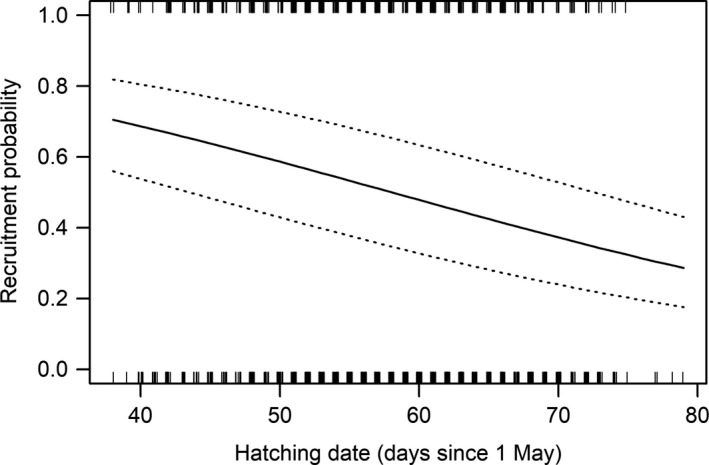
Variation in the probability (solid line ± *SE*, dashed lines) of Icelandic godwit chicks recruiting to the wintering or subsequent breeding population in relation to their hatch date

For Icelandic godwit chicks, the probability of successfully recruiting to the wintering population is strongly influenced by hatch date (Table 1a). Chicks hatched during the first two weeks of June (before day 47 on Figure 3) have, on average, >60% probability of recruiting, whereas those hatching after 12 July (day 73 on Figure 3) have, on average, <35% probability of recruiting. The benefits of early laying in warmer years could be reduced if hatching success was lower early in the season, but we found no seasonal effect of laying date on daily nest survival (Table 1b). In colder years (2001, 2011), when average June temperatures were below 9.5°C, the mean percent of chicks hatched before day 57 (when recruitment probability exceeds 50%, Figure 3) was 45% (± 1 *SE*). By contrast, in warmer years (average June temperature≥11°C; 2010, 2012), an average of 64% (± 5 *SE*) of chicks hatched early (before day 57), indicating the substantial effect that early hatching can have on the overall numbers of chicks recruiting from warmer than colder years.

**Table 1:** Results of binomial GLMMs of: (a) the influence of hatching date on the probability of Icelandic black‐tailed godwit chicks recruiting to the wintering population; (b) the effect of laying date on daily nest survival probabilities

	Estimate	*SE*	*z* value	*p*
(a)				
Intercept	2.53	0.62	4.05	**<0.001**
Hatch date	−0.04	0.01	−4.55	**<0.001**
(b)				
Intercept	−2.71	0.46	−5.9	**<0.001**
Lay date	−0.01	0.01	−0.55	0.58

Annual variation (in a and b) is included as random factor (see methods for details). Significant effects are highlighted in bold.

### 3.3 The influence of temperature, phenology and demography on range expansion

**Figure 4:**
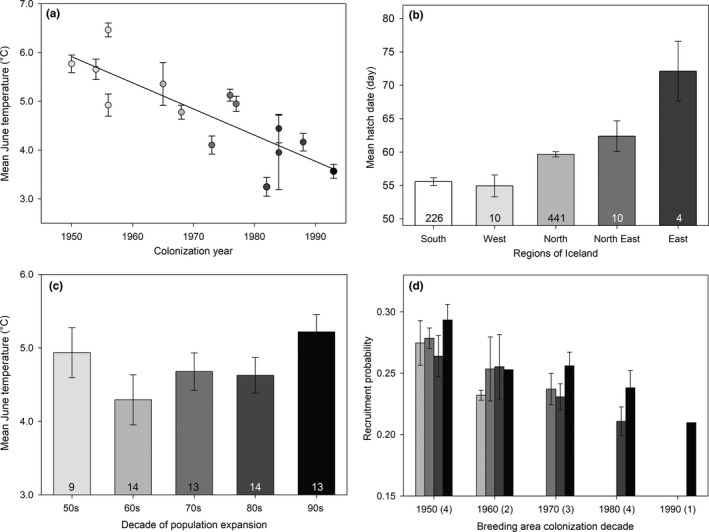
Variation in mean (± *SE*): (a) June temperature in 14 lowland areas in Iceland colonized by godwits during the second half of the 20th century (lowland areas colonized in different decades colored as in Figure 1; see Supporting Information Table S3 for details of temperature ranges); (b) hatch dates (ordinal days since 1st of May) of godwit chicks between 1999 and 2011 across regions of Iceland for which temperature and colonization dates are available (number of chicks per region given within bars and bar color reflects the predominant decade of colonization within each region); (c) June temperatures in five decades during the range expansion period (see Table 2 for statistics); and (d) the potential consequences of spatial and annual variation in June temperatures and chick hatch dates for estimated annual recruitment probability in areas colonized in different decades (number of areas with available temperature data shown in parentheses, details in Supporting Information Table S3) during the 1960s (light gray bars), 1970s (gray bars), 1980s (dark gray bars), and 1990s (black bars) across Iceland

The sequence of colonization of lowland areas around Iceland by breeding godwits over the last century follows a temperature gradient, with warmer areas being colonized first (Table 2; Figure 4a), and godwit chicks hatch earlier in regions that were colonized first (*F*_4,686_ = 12.8, *p* < 0.001; Figure 4b; Supporting Information Table S3). Following relatively warm average June temperatures in the nine areas around Iceland with available temperature data in the 1950s (and when few sites had been colonized; Figure 1), subsequent temperatures were lower in the 1960s but have generally increased since then (Table 2; Figure 4c).

**Table 2:** Results of a GLM of variation in mean June temperatures over five decades (1950s–1990s) in 14 breeding areas around Iceland colonized in different years

	*df*	F	*p*	Estimate (± *SE*)
Colonization year	1	81.66	**<0.001**	−0.06 (±0.01)
Decade	4	3.88	**0.008**	
Col. year*Decade	53	0.68	0.609	
Error	62			

Significant effects are highlighted in bold.

The changes in June temperatures around Iceland since the 1950s (Figure 4a,c) can be used to assess the potential magnitude of temperature‐driven variation in hatch dates and recruitment of Icelandic godwits. In every decade, the areas of Iceland that were colonized during the 1950s were warmest and estimated recruitment is therefore consistently highest in these areas (Figure 4d). In the 1990s, when all areas had been colonized, recruitment probability was estimated to be highest in the earlier occupied (warmer) areas and lowest in the most recently colonized (colder) areas (Figure 4d—black bars). Rapid warming between the 1980s and 1990s (Figure 4c) could potentially therefore have driven a widespread increase in recruitment rates of up to ~3.0% (Figure 4d) across most of lowland Iceland during this later decade (with the exception of areas colonized during the 1960s, for which temperature data in the 1990s are only available for one site). Warming temperatures facilitating earlier nesting and associated increases in chick recruitment therefore have the potential to have contributed quite substantially to the post‐colonization population growth around Iceland.

## 4 DISCUSSION

Phenological changes in response to changing climatic conditions have been widely reported across many taxa in recent years, but the evidence for population‐level consequences of these shifts for free‐ranging species varies considerably (e.g., Both et al., 2006; Wright et al., 2009). Although climate‐driven changes in phenology may have little effect on population‐level processes in some cases (Dunn & Møller, 2014; Reed, Grøtan, et al., 2013a), phenological changes that influence demography have the potential to alter population and range size, particularly if these changes facilitate local population growth and range expansion into previously unoccupied areas in which density‐dependent constraints may be weak (McLean et al., 2016). Icelandic black‐tailed godwits have colonized and become established in breeding locations around lowland Iceland over the last century, concomitant with decades of sustained warming. In south Iceland, earlier nesting occurs in years with warmer temperatures, resulting in advances in hatching dates. As early hatching confers substantial increases in chick recruitment probability, the widespread temperature increases around Iceland may have contributed substantially to the sustained population increase and range expansion of Icelandic godwits during the last century, if the temperature effects on breeding phenology occur throughout the range. As godwits conceal their nests from predators in tall vegetation, more rapid vegetation growth in recent decades in response to warming could have facilitated earlier (and perhaps more successful) nesting across the breeding range. Given the consistently high adult survival rates and philopatry of godwits (Alves et al., 2013; Gill et al., 2001; Gunnarsson, Sutherland, Alves, Potts, & Gill, 2012), increases in recruitment rates as a consequence of earlier nesting are likely to have contributed substantially to the post‐colonization establishment and growth of local populations around Iceland.

### 4.1 Effect of temperature on timing of breeding season events

The timing of nest laying and the length of the pre‐fledging period varied in relation to temperature, with warmer conditions being associated with earlier and shorter breeding periods. In this system, mean laying dates varied by ~11 days between the warmest (11.2°C, estimated laying = 29.2 ± 2.7 days) and coldest years (9.2ºC, estimated laying = 40.2 ± 2.7 days), and this was the dominant driver of annual variation in mean hatching dates. In contrast, the lengths of pre‐fledging period varied by ~3.6 days between the warmest and coldest years. The annual variation in nest laying dates could be also influenced by carry‐over effects of adult pre‐breeding condition, nesting site availability, or suitability of conditions for chick‐rearing. Icelandic godwits are known to migrate with sufficient reserves (Alves et al., 2012) and typically arrive in Iceland 2–3 weeks before nest initiation (Gill et al., 2014). This substantial amount of time to improve body condition prior to nesting may reduce the effects of any carry‐over from winter conditions (Harrison, Blount, Inger, Norris, & Bearhop, 2011). However, faster vegetation growth rates in warmer springs may facilitate earlier nesting because Icelandic godwits are among the largest‐bodied nest‐concealing species of their family (Cramp & Simmons, 1983) and, while average vegetation height in the vicinity of godwit nests on our study areas was ~9–10 cm by mid May, godwit nests have on average a vegetation height of 23.3 cm (±1.2 *SE*,* n* = 45). The spring onset and rate of growth of the grasses and sedges in these wetlands are positively influenced by warm temperatures, particularly by the length of warm periods (Thorvaldsson et al., 2005). Thus, in years with warmer average temperatures, vegetation will reach suitable heights faster, removing potential limitations on nesting site availability (i.e., patches with tall enough vegetation to conceal nests), which may allow godwits to nest earlier than in colder years. In addition to suitable vegetation heights for nesting, food resources for chicks are also likely to be more abundant earlier (and generally) in warmer years (Tulp & Schekkerman, 2008), and this association may also contribute to the success of early breeding attempts.

The pre‐fledging period of godwits was slightly longer in colder temperatures. Variation in the length of the pre‐fledging period is a function of chick growth rate, which is also influenced by temperature, with the sum of daily temperature during the pre‐fledging period likely to provide a proxy for chick foraging time. At low ambient temperatures, wader chicks need to be brooded by their parents for longer (Schekkerman & Boele, 2009), which can limit foraging opportunities and result in depressed growth rates and, in extreme cases, death by starvation (Beintema & Visser, 1989). Greater abundance of invertebrate prey resources for chicks in warmer conditions (Halldórsson et al., 2013) is also likely to translate into faster chick growth rates (Eglington et al., 2010). In addition, the trade‐off in allocating resources between somatic growth and body temperature (Price & Dzialowski, 2018) is likely more biased toward the latter in colder years.

### 4.2 Cascading effects of temperature‐mediated phenological changes on demography and distribution

Overall, the additive effects of an ~11‐day difference in timing of laying, and a ~3.6‐day difference in pre‐fledging period can result in an average difference of up to ~14.6 days at fledging between warm (~11°C) and cold (~9°C) years. The ~11‐day delay in hatch dates resulting solely from laying dates in warm and cold years equates to a difference in recruitment probability of ~10% (Figure 3), with the additional benefits of earlier fledging in warmer years likely contributing to the higher probability of recruitment. The decline in recruitment probability with hatch date means that in cold years, when most nests are laid late, very few chicks are likely to recruit to the adult population. For example, in 2011, the coldest year recorded during our study, only ~10% of 101 ringed chicks recruited into the wintering population. Benefits of early hatching for recruitment are likely to be manifest through advantages associated with successful and early fledging, such as increased time to improve body condition prior to migration, increased probability of traveling in adult‐dominated migratory flocks, and earlier departure for winter grounds allowing more time in which to select a favorable wintering location (Alves et al., 2013; Gunnarsson, 2006). Given that adult survival is relatively high (0.91 ± 0.02 *SE*) and with little annual variability (Alves et al., 2013), recruitment into the adult population is likely to be a major driver of population growth. Positive associations between temperature and productivity have been reported in migratory common sandpiper *Actitis hypoleucos* but these appear to have been offset by declines in adult survival rates (Pearce‐Higgins, Yalden, Dougall, & Beale, 2009).

Iceland's position in the North Atlantic, between the Atlantic and Arctic oceans, means that the south and west of the country typically experience warmer temperatures than the north and east. Over the last century, the godwit breeding range has expanded from the warmer south and west regions to colonize new sites in the north and east (Gunnarsson, Gill, Petersen, et al., 2005b). Estimated recruitment probability is consistently higher in traditionally occupied areas (Figure 4d), primarily as a consequence of the earlier laying and hatching that is possible in these warmer areas (Figure 4b), and potential additional benefits of early fledging. The estimations of recruitment probability assume that the relationships between temperature and timing of breeding season events persist at lower temperatures than those recorded on our study sites. These assumptions are likely to be conservative, as colder conditions may well delay nesting even more than a linear extrapolation of Figure 2a, and it is extremely unlikely that laying dates would advance at such temperatures. Population size estimates for Icelandic godwits on the wintering grounds are available for the United Kingdom since the late 1970s, through the British Trust for Ornithology Wetland Bird Survey. The lack of change in estimated recruitment rates between the 1970s and 1980s, followed by widespread increases from the 1980s to the 1990s, matches counts on the UK wintering grounds, where a period of stability in the 1980s was followed by a sustained increase in numbers during the 1990s (Holt et al., 2015), again suggesting a contribution of warming‐driven changes in recruitment to the population expansion.

Increased recruitment in new areas could also be influenced by increases in food resources or habitat availability, or by reduced levels of nest predation. During the early 19th century, both avian and terrestrial nest predator species increased in Iceland, with gull (*Larus fuscus* and *L. argentatus*) and owl (*Asio flameus*) species colonizing the country in ~1920 to 1930 and increasing in numbers until ~1990 to 2010 (Skarphéðinsson, Katrínardóttir, Guðmundsson, & Auhage, 2016), and mink (*Mustela vision*) spreading around the country after escaping captivity in 1937 (Bonesi & Palazon, 2007). Population trends of native nest predators as raven (*Corvus corax*) and arctic skua (*Stercorarius parasiticus*) are unknown, but arctic foxes (*Vulpes lagopus*) which declined in the 1960s, have increased since to numbers above those recorded in the late 1950s (Unnsteinsdottir, Hersteinsson, Pálsson, & Angerbjörn, 2016). For species breeding at the northern and colder areas of the distribution range, warming temperatures likely relax environmental constraints (e.g., vegetation growth, food abundance) and can positively influence productivity and population growth. The range expansion in this system could have been driven by increased productivity and continuous dispersal from traditionally colonized areas and/or by increased productivity within newly colonized areas. Given the high levels of natal philopatry in waders, and specifically in this population (Gunnarsson et al., 2012), it seems likely that improved breeding conditions following colonization of these areas have contributed substantially to the population increase, particularly given the consistently high adult survival rates in this population in recent decades (Alves et al., 2013). Although some recent studies report that climate‐driven changes in phenology might not always lead to population‐level consequences (Dunn & Møller, 2014; Reed, Jenouvrier, et al., 2013b), in Icelandic godwits the links between temperature effects on breeding phenology and subsequent juvenile recruitment may be apparent because they have occurred during a phase of population expansion and colonization of previously unoccupied breeding areas, in which density‐dependent constraints on growth are likely to have been relatively weak (McLean et al., 2016).

Identifying the mechanisms through which environmental changes influence population size and distribution is key to predicting the future status of species and designing appropriate conservation strategies (McLean et al., 2016). The phenological changes that are being widely recorded across taxa at present have the potential to influence fitness and may therefore be a key component of many of the demographic and range shifts being reported. Projections of future distributions of species rely largely on associations between their current distribution and the climatic conditions that they experience, but these associations are often weak (Beale et al., 2008), and lack the mechanistic basis required for robust predictions (Norris, 2004). Understanding relationships between phenology and fitness, the environmental processes that underpin these relationships, and the conditions in which these changes can influence population dynamics and distribution are likely to greatly improve projections of future distributions of species.

## CONFLICT OF INTEREST

None declared.

## AUTHORS' CONTRIBUTIONS

JAA, JAG, TGG and WJS formulated the idea and designed the research program. JAA, TGG, JAG and PMP collected and compiled the data. JAA developed the analysis with support from JAG. JAA, JAG and TGG wrote the article with contributions from WJS.

## Data Availability

All data used in this manuscript are presented in the figures and supporting information.
