# Supplementary material for: Linking warming effects on phenology, demography, and range expansion in a migratory bird population
Source: Ecol Evol. 2019 Feb 14;9(5):2365–75. doi: 10.1002/ece3.4746 (PMC6405501; doi:10.1002/ece3.4746)
Supplement: Supplementary file 1 [file ECE3-9-2365-s001.docx]

**Supporting Information**

*Alves et al. “Linking warming effects on phenology, demography and range expansion in a migratory bird population.”*

*Estimating recruitment probability during range expansion*

Using mean decadal June temperatures for each lowland area (Table S3) we firstly estimated laying date (Ld) from Eqn. 1 (Fig. 2A). By summing the estimated laying date (Eqn. 1) and the 23 day incubation period (Table S2) we estimated mean decadal hatch date (Hd), which was then used to estimate recruitment probability (Eqn. 2) from the model presented in Fig. 3 and Table 1A.

Eqn. 1: Ld = - 5.49Jt + 90.7

Eqn. 2: Rp = 1/1^-(2.53-0.049×Hd)^

where Ld = Laying date, Jt = decadal mean June temperature, Rp = Recruitment probability and Hd = Hatching date.

**Table S1.** Number of Icelandic black-tailed godwit nests and families tracked across 11 field-sites in the Southern Lowlands of Iceland.


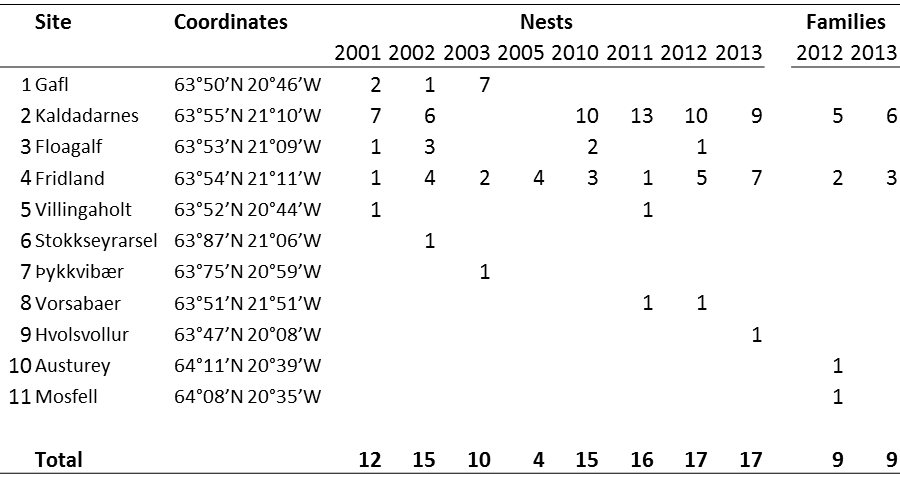


**Table S2.** Egg floatation angle and height adapted from Liebezeit *et al.* (2007) for Icelandic godwits. Estimated number of days to hatch is given for a total incubation period of 23 days (average days to hatch from nests found at incubation stage 1 = 22.7 ± 0.3 (SE), n = 7).


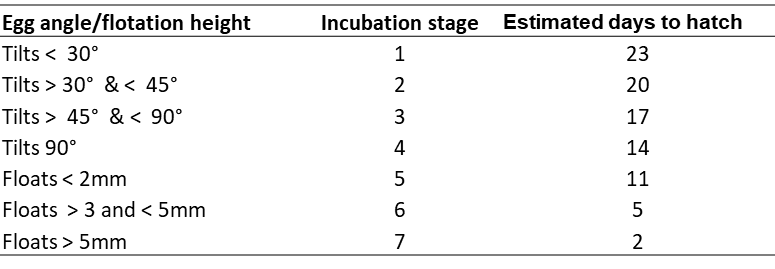


**Table S3.** Average year of colonisation by Icelandic godwits of 14 lowland areas in Iceland (the Southern Lowlands was the only area occupied at the start of the 20^th^ century), and details of the representative manned weather recording stations located within the corresponding lowland basin from which temperature data were extracted for each area (available at www.vedur.is). Only one weather station (Hvanneyri) does not cover the colonisation period of the area.


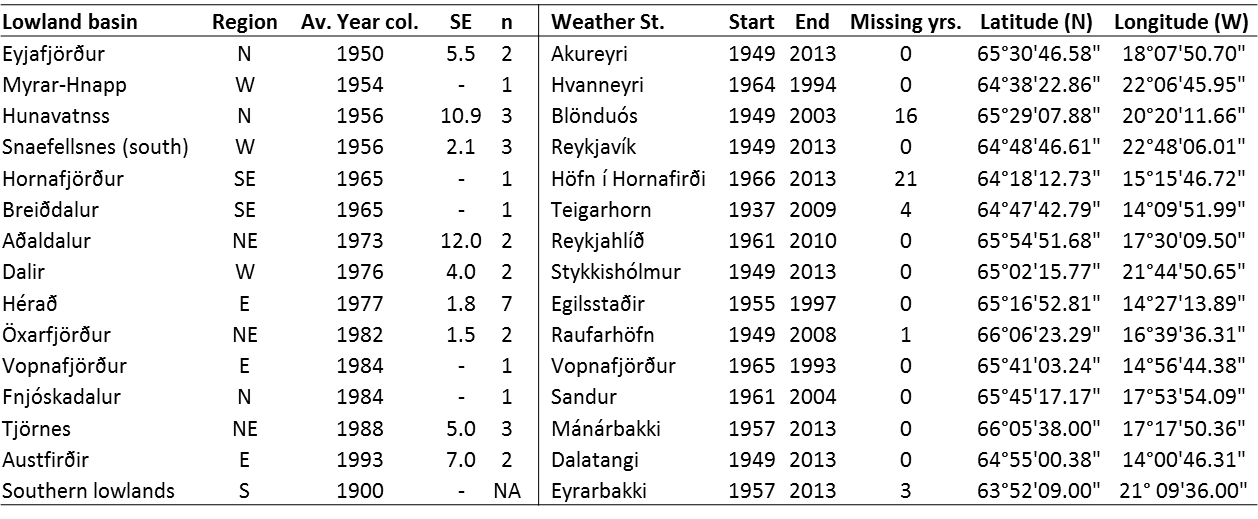


***Note to Table S3:*** To estimate the year of colonisation, Gunnarsson *et al.* (2005b, in main text) collated records from the Icelandic Institute of Natural History and published sources to date the major patterns of colonisation of breeding sites around Iceland by black-tailed godwits since the 1900s. In total, 118 records of first or recent breeding were obtained from these sources.
